# A Will Contested on the Plea of the Insanity of the Testator

**Published:** 1848-07-01

**Authors:** 


### A WILL CONTESTED ON THE PLEA OF THE INSANITY OF THE TESTATOR.

The following abstract possesses some interest, as showing the law and practice on this subject in France.

M. Vicquelin, an ancient counsellor of the royal court of Rouen, executed a will in his own hand-writing, by which he bequeathed all the property which the law allowed him to dispose of, to collateral relatives, but reserved the income of the same to his daughter, Dame Levacher. She applied for a declaration of the nullity of the will, on the ground that before, during, and after its execution, the testator was of unsound mind, and she offered to prove the same by his writings, and also by the testimony of competent witnesses.

The case was dismissed by the inferior tribunal, and she now appealed to the royal court of Rouen. The attorney-general opposed it on the following grounds:—

The mental capacity of M. Vicquelin was never judicially disputed during his lifetime. The presumption is therefore in favour of the will. This may, however, be invalidated by sufficient testimony. But there should be proof of habitual insanity; and if this be established, it will not be necessary to show its presence precisely at the time of making the will. Present evidence of its existence before and after, and the defenders of the testamentary provisions must prove a lucid interval. But until the existence of insanity be thus shown, the will must stand, particularly as there is nothing extravagant in its dispositions.

In monomania the lucid interval is its disappearance. The sane acts of a monomaniac do not constitute a lucid interval, as it is the essence of the disease to be reasonable on many points disconnected with the prevailing delusion.

If the insanity proceeds from a weakness of mind, produced by age or disease, there also will be lucid intervals! Under such circumstances, one and the other alternate, and the presence of reason constitutes in this instance the lucid interval. If it be altogether absent, the individual verges to idiocy.

When we proceed to apply these abstractions to the case before us, we find that there is no charge of monomania. The insanity specified by the appellant is mental imbecility—the conjoint effect of age and disease. And here, all reasonable acts proved to have been performed by M. Vicquelin, of his own free will, and at his own instance, without persuasion or solicitation, are proofs of the occurrence of a lucid interval.

Nor must we confound the occurrence of anger or rage, however frequent, with mania. Allowance must also be made for oddities of character and peculiarities of mind. We consider Sepoitevin as insane, because he went to the Tuilleries dressed in a helmet, and with the cordon of the Legion of Honour, and mistook Madame Adelaide for the king, and addressed her in incoherent language. So also with the person who, fifteen years after the death of his wife, entered a complaint of adultery against her before a justice of the peace. Such conduct does not admit of more than one construction, but the accidental occasional loss of memory is very different, otherwise we must include La Fontaine in the number of the insane, because he hissed a performance at the theatre, of which he forgot that he himself was the author.

The avocat-général then proceeded to consider the will itself and its provisions, showing that it was the result of his own free wishes, uninfluenced by any suggestion or urging, while at the period of making it, it was proved that he was constantly consulted by his neighbours on business matters. The letters written by him, or dictated, and, indeed, his whole correspondence, proved that his intellect was unaffected. It was only when suffering under severe attacks of pain that he was guilty of violent or unreasonable actions.

The court decided in favour of the will. They state that the testator had laboured from March, 1840, to the period of his death, (January 31, 1842,) under an almost incessant nervous irritation, caused by accidents that had impaired his health, and while under its influence, frequently yielded to fits of anger and violence, and, indeed, committed many acts which might be styled extravagant. But although these might throw a shade on his sanity, yet when it is remembered that during the same period, and even in the last month of his life, many persons, and even public officers, consulted him on their affairs; that he had written many opinions with his own hands; that he had managed his property with minute attention; and that, although there were proofs of puerility in the last days of his life, still his letters, notes, and memoranda, by their number, precision, and good sense, indicate that his mind had resisted the attacks of age and disease so far, at least, as concerned his own property.

These considerations acquire additional force by the proof that these numerous writings were executed by the testator at the time of making the will, as well as immediately before and after.

But the will itself is the best proof of his sanity, in its form, style, quotations from the law, and minute enumerations. All these were the sole and unaided work of M. Vicquelin, and expressed with such force and clearness, that even if we grant the occasional presence of imbecility, the paper itself must be conceded to have been the product of a lucid interval.—*Gazette des Tribunaux.*

